# Association of Chromosome 17 Aneuploidy, *TP53* Deletion, Expression and Its rs1042522 Variant with Multiple Myeloma Risk and Response to Thalidomide/Bortezomib Treatment

**DOI:** 10.3390/cancers15194747

**Published:** 2023-09-27

**Authors:** Sylwia Popek-Marciniec, Wojciech Styk, Magdalena Wojcierowska-Litwin, Sylwia Chocholska, Aneta Szudy-Szczyrek, Marzena Samardakiewicz, Grazyna Swiderska-Kolacz, Joanna Czerwik-Marcinkowska, Szymon Zmorzynski

**Affiliations:** 1 Laboratory of Genetics, Academy of Zamosc, 22-400 Zamosc, Poland; sylwia.popek@gmail.com; 2 Department of Psychology, Medical University of Lublin, 20-059 Lublin, Polandmarzena.samardakiewicz@umlub.pl (M.S.); 3 Department of Cancer Genetics with Cytogenetic Laboratory, Medical University of Lublin, 20-059 Lublin, Poland; 4 Chair and Department of Hematooncology and Bone Marrow Transplantation, Medical University of Lublin, 20-059 Lublin, Polandanetaszudy@gmail.com (A.S.-S.); 5 Institute of Biology, Jan Kochanowski University, 25-369 Kielce, Poland; grazyna.swiderska-kolacz@ujk.edu.pl (G.S.-K.); marcinko@kielce.com.pl (J.C.-M.)

**Keywords:** *TP53* variant, p.P72R polymorphism, *TP53* expression, 17p13 *locus*, plasma cell myeloma, plasma cells

## Abstract

**Simple Summary:**

The detailed role of the *TP53* p.P72R (rs1042522) variant in the etiology and course of multiple myeloma (MM), as well as its association with chromosome 17 aberrations, has not been analyzed in a such a wide range. In our study, we have explored the significance of the p.P72R variant, expression level of *TP53* gene, deletion of *TP53 locus* and chromosome 17 aneuploidies in MM development, and the response to bortezomib/thalidomide treatment in MM patients. We have shown that the p.R72R variant was associated with a higher age of MM patients at diagnosis and with a higher number of plasma cells. We have found higher expression of *TP53* in MM smokers in comparison to MM non-smokers. *TP53* gene expression and the p.P72R variant did not affect the MM outcome.

**Abstract:**

Multiple myeloma (MM) is a multifactorial genetic disorder caused by interactive effects of environmental and genetic factors. The proper *locus* of the *TP53* gene (17p13.1) and its protein is essential in genomic stability. The most common variant of the *TP53* gene—p.P72R (rs1042522)—shows functional variation. The aim of our study was a complex analysis of the *TP53* p.P72R variant and *TP53* gene expression in relation to chromosomal changes of the *TP53* gene *locus*, as well as MM risk and outcome. Genomic DNA from 129 newly diagnosed MM patients was analyzed by methods of automated DNA sequencing (for *TP53* variant analysis) and cIg-FISH (for chromosomal aberrations analysis). RNA was used in real-time PCR to determine the *TP53* expression. In MM patients, the *TP53* variant was not in Hardy–Weinberg equilibrium. The RR genotype was associated with lower MM risk (OR = 0.44, *p* = 0.004). A higher number of plasma cells was found in patients with RR genotype in comparison to those with PP + PR genotypes (36.74% vs. 28.30%, *p* = 0.02). A higher expression of the *TP53* gene was observed in PP + PR genotypes vs. RR homozygote (*p* < 0.001), in smokers vs. non-smokers (*p* = 0.02). A positive Pearson’s correlation was found between the *TP53* expression level and the number of plasma cells (r = 0.26, *p* = 0.04). The presence of chromosome 17 aberrations with or without *TP53 locus* did not affect the MM risk and outcome. Similar results were observed in the case of *TP53* gene expression and the p.P72R variant.

## 1. Introduction

Multiple myeloma (MM) is a hematological malignancy, characterized by the proliferation of abnormal plasma cells [1,2]. MM is caused by genomic changes in the form of point mutations and chromosomal aberrations. The latter include translocations, deletions and duplications present in DNA of plasma cells [3,4]. Among the listed changes, deletion of the region containing *TP53* gene is important in MM prognosis.

The *TP53* gene is located on chromosome 17 (*locus* 17p13.1) and encodes p53 (P53, TP53) protein enhancing transcription of its target genes [5]. The *TP53* gene functions as a tumor suppressor. It consists of 11 exons and its product acts as a transcription factor regulating cell cycle progression and cell growth. Normal TP53 protein maintains genomic integrity, inhibits angiogenesis and carcinogenesis [5,6]. The loss and/or mutation of the *TP53* gene is associated with a shorter overall survival (OS) in MM [7]. At the MM diagnosis point, mutations of the *TP53* gene are uncommon and are less than 8% of all cases [7]. Deletion of the chromosomal region containing the *TP53* gene (17p13.1) has been identified in about 5–10% of newly diagnosed MM cases [7]. Loss of the 17p13.1 *locus* is associated with poor prognosis of MM [8,9]. The prognosis is poorer when the clonal fraction of del(17)(p13.1) is higher than 60% in all myeloma cells [10]. Below this level, the prognosis is not significantly affected [4,10]. However, currently used conventional methods for detecting 17p13.1 aberrations underestimate their incidence, complicating the optimal risk assessment and prognosis of patients with MM [8,11,12].

Deletions of 17p13.1 are associated with a low expression level of the p53 protein [13]. Moreover, a low *TP53* gene expression level is associated with inferior outcome in various types of cancers, including breast cancer, acute myeloid leukemia, and squamous cell carcinoma of the head and neck [14,15,16]. The coexistence of both *TP53* deletions and mutations, which define the so-called double-hit patients, is associated with the worst clinical outcome [8,17]. In addition, the allelic status of *TP53* may change during the course of MM. The acquisition of *TP53* changes during relapse dramatically worsens the clinical course of patients [17].

Some variants of the *TP53* gene with clinical significance have been described. Most polymorphisms present in the *TP53* gene are innocuous, but some may have clinical significance in cancer progression and response to treatment [18]. The latter group includes the *TP53* variant (rs1042522), which is present in codon 72 (exon 4) (p.P72R) [19,20]. Meta-analysis performed by Akhtar and Alharbi found a significant association between the p.P72R (Pro72Arg) polymorphism and leukemia susceptibility [21]. This variant is a type of single nucleotide polymorphism (SNP) and is associated with the presence of a nucleotide with G or C leading to codon transition of CGC (for arginine) to CCC (for proline) [19,20]. As a result of substitution, amino acid proline (Pro, P) is present in the protein chain instead of arginine (Arg, R) in the proline-rich domain (PRD) [19,20]. PRD is required for the cooperation with anti-tumor agents to initiate apoptosis [22]. According to the National Center for Biotechnology Information, the rs1042522 variant in the European population is associated with the presence of a reference allele (wild-type allele, R allele, 72R)—encoding Arg—and alternative allele (P allele, 72P)—encoding proline [23].

In the *TP53* gene mutations are present regardless of the p.P72R variant. Immunohistochemistry results showed a correlation between the p53 protein’s mutations and its overexpression [5]. Yemelyanova et al. found that the immunohistochemical pattern of p53 in tumors is a predictor of *TP53* mutations [24]. Mutated p53 protein with p.72R induced the expression of genes associated with cell proliferation and pro-oncogenic pathways [20]. The R allele is associated with an increased risk of solid tumors development, such as colorectal cancer, breast cancer and lung cancer [25]. In contrast, the allele encoding Pro (P allele) causes more efficient cell cycle arrest in the G1 phase, and induces DNA repair mechanisms and the endoplasmic reticulum stress response [26,27]. The presence of the P allele increases the development risk of lung and thyroid cancers [28]. The presence of the P allele is associated with a tumor-suppressor role of p53, whereas the R allele determines the tumor-promoting role for this protein [20]. Taking into account that in tumor suppressor genes are present loss-of-function mutations and in proto-oncogenes, gain-of-function changes, we assume that the presence of the p.P72R variant may affect *TP53* gene expression. Differential expression of *TP53* in various types of cancers may be determined by p.P72R variant [29].

We hypothesize that the *TP53* variant, expression level of *TP53* and its deletion may be associated with a higher risk of MM development and may also affect the response to treatment of MM patients. This study investigates *TP53* changes and their association with selected clinical and laboratory disease parameters, and the response to bortezomib/thalidomide-based therapies.

## 2. Materials and Methods

### 2.1. Patients

The study enrolled 229 unrelated individuals, including 129 newly diagnosed MM patients and 100 healthy blood donors. The study participants were selected from the same ethnicity (Caucasian population). Material from the Chair and Department of patients between 2013 and 2020 was provided by clinicians from the Chair and Department of Hematooncology and Bone Marrow Transplantation (Medical University of Lublin, Poland). The characteristics of MM patients are presented in Table 1.

The control group (for genotyping analysis) consisted of healthy blood donors (50 men and 50 women, with a mean age of 37.6 years) from the Regional Blood Donation and Blood Treatment Center in Kielce, Poland.

For the expression study as a control group, bone marrow aspirates obtained from 18 non-neoplastic patients with orthopedic injuries hospitalized at Department of Orthopedic and Trauma Surgery of Jan Kochanowski University of Kielce were used. From bone marrow aspirates, nucleated cells were isolated to carry out gene expression analysis. In this regard, plasma cells were isolated from MM bone marrow aspirates with the use of a magnetic method with CD138+ beads, according to the manufacturer’s protocol (Miltenyi Biotec, Bergisch Gladbach, Germany). From bone marrow aspirates obtained from non-neoplastic patients with orthopedic injuries, mononuclear cells were insolated with use of Lymphoprep (Serumwerk, Bernburg, Germany).

The study obtained positive opinions from the Bioethics Committee at the Medical University of Lublin (No. KE-0254/165/2013, No. KE-0254/337/2016) and the Bioethics Committee at Jan Kochanowski University of Kielce (No. KB-41/2016), according to the ethical standards established by the Helsinki Declaration. All methods were performed in accordance with the relevant guidelines and regulations. All study participants provided written informed consent. The inclusion and exclusion criteria for all individuals in the study are described in Table 2.

### 2.2. DNA Isolation

DNA was isolated from the peripheral blood of healthy blood donors and MM patients. For this purpose, a commercial kit (Qiagen, Velno, The Netherlands) was used according to the manufacturer’s recommendations. The concentration and quality of the obtained nucleic acid were then checked using a NanoDrop device (Thermo Fisher Scientific, Waltham, MA, USA).

### 2.3. TP53 Genotyping

**Figure 1:**
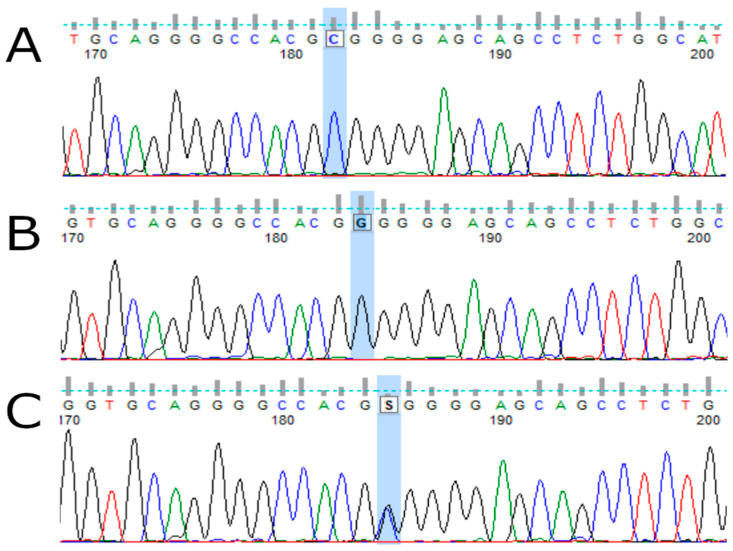
Sample electrophoregrams showing individual variants of the rs1042522 polymorphism of the *TP53* gene. (**A**)—Pro72Pro (GCG) variant, (**B**)—Arg72Arg (GGG) variant, (**C**)—Pro72Arg variant.

DNA obtained from peripheral blood samples (of MM patients and healthy blood donors) was used to determine the *TP53* codon 72 polymorphism. The *TP53* rs1042522 status was determined by automated sequencing according to the protocol shared by the International Agency of Research on Cancer (IARC). A fragment of exon 4 (of the *TP53* gene) was amplified by PCR (Applied Biosystems 9700 Thermal Cycler) using primers: forward 5′-TGC TCT TTT CAC CCATCT AC-3′; reverse 5′-ATA CGG CCA GGC ATT GAA GT-3′. Each PCR mixture (15 µl) contained 50 ng of genomic DNA, Color Perpetual Taq PCR Master Mix (EurX, Gdansk, Poland) and primers (10 µM of each). The mixture was heated at 94 °C for 2 min and then the first 20 amplification cycles occurred as follows: denaturation at 94 °C for 30 s, annealing at 63 °C (−0.5 °C every 3 cycles) for 45 s, elongation at 72 °C for 1 min and 30 subsequent amplification cycles under the conditions of denaturation at 94 °C for 30 s, annealing at 60 °C for 45 s, and elongation at 72 °C for 1 min. The final elongation took 10 min at 72 °C. Sequencing PCR and analysis of the results were prepared as previously described [30]. The results were analyzed with the use of Applied Biosystems software—Data Collection Software version 3.1 (Figure 1).

### 2.4. Cytoplasmatic Immunoglobulin and FISH (clg-FISH) Method

Chromosomal aberrations with prognostic significance in MM were tested using cytoplasmic immunoglobulin and FISH (cIg-FISH) in accordance with the recommendations of Ross et al. 2012 [31]. Culture and staining of malignant plasma cells were performed according to the previously described protocol with modifications [32,33]. The results’ analysis with the used probes was carried out as previously described [34].

### 2.5. RNA Isolation and TP53 Expression

RNA isolation from mononuclear bone marrow cells was carried out with use of total RNA midi kit (Aabiot, Gdansk, Poland). The concentration of isolated RNA was checked spectrophotometrically using a NanoDrop device (Thermo Scientific, Waltham, MA, USA). The quality of this nucleic acid was checked during electrophoresis in 2% agarose gel. RNA was stored at −80 °C.

The reverse transcription reaction (RT-PCR) was prepared after RNA isolation. RT-PCR was performed using a 9700 Thermal Cycler (Applied Biosystems, Waltham, MA, USA). The real-time PCR reaction (Applied Biosystems 7500 Fast) was carried out on the cDNA (100 ng) template. Real-time PCR was performed using the SYBR Green RT-PCR Mix (Aabiot, Poland), an annealing temperature of 55 °C, and 100 μM primers (Genomed, Warszawa, Poland): forward 5′-TTG CCG TCC CAA GCA ATG GAT GA-3′, reverse 5′-TCT GGG AAG GGA CAG AAG ATG AC-3′.

The 20 µL of the reaction mixture was transferred to each well of the PCR reaction plate. The plate was centrifuged and placed in a 7500 Fast Real-time PCR apparatus (Applied Biosystems, Waltham, MA, USA). The qRT-PCR reaction was prepared according to the manufacturer’s protocol (Aabiot, Gdansk, Poland). Every sample was assayed in duplicate with expression being calculated according to the 2^−ΔΔCt^ method—normalized expression [35]. Expression values were presented as the logarithm of R to the base 2, where R was calculated as follows: R = 2^−ΔΔCt^, ΔΔCt = ΔCt of control −ΔCt of analyzed gene, every ΔCt = Ct of analyzed gene − Ct of endogenous control. The expression of GADPH served as a control. For this purpose, 100 μM primers (Genomed, Warszawa, Poland) were used with annealing temperature of 60 °C: forward, 5′-CAACGGATTTGGTCGTATTG-3′; reverse, 5′-GGATCTCGCTCCTGGAAG-3′.

The value of the normalized expression (R) in the range of 0.8–1.2 indicated the normal level of gene expression, R < 0.8 indicated low expression, and R > 1.2 higher expression [36,37].

### 2.6. Statistical Analysis

Laboratory results of MM patients were compared with the studied *TP53* gene genotypes using the *t*-test (for continuous variables) and the Chi-square test (for categorical variables). The association between *TP53* genotypes and clinical data was assessed using the Chi-square test or Fisher’s exact test (when one expected value was <5). Quantitative data were presented as frequency or percentage. The Hardy–Weinberg equilibrium (HWE) was assessed using the Chi-square test with Yates correction for groups of less than five individuals. For the 95% confidence interval (CI), we assumed *p* = 0.05 and χ^2^ = 3.84; thus, if χ^2^ ≤ 3.84 and corresponding *p* ≥ 0.05, then the population is in HWE. The Cox proportional hazard model was used for univariate and multivariate analysis of OS and progression-free survival (PFS). The Kaplan–Meier method and log-rank test were used for survival analysis. Pearson correlation analysis was used to evaluate the relationship between *TP53* expression level and laboratory/clinical data. We assumed a 5% error of inference and an associated significance level of *p* < 0.05 indicating the existence of statistically significant differences. Statistical analyses were performed using Statistica ver. 12.5 (StatSoft, Krakow, Poland).

## 3. Results

**Figure 2:**
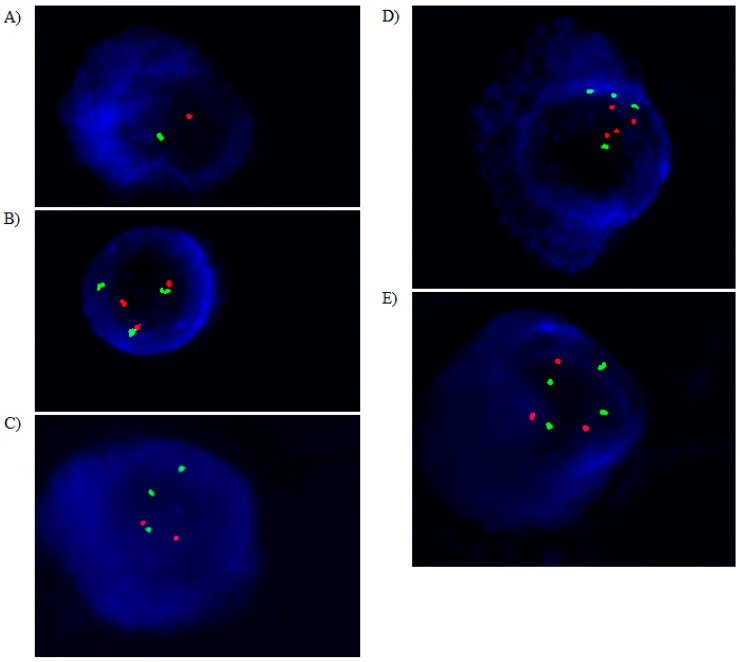
Simultaneous staining of cytoplasmic immunoglobin and FISH (cIg-FISH): (**A**) chromosome 17 monosomy; (**B**) chromosome 17 trisomy; (**C**) chromosome 17 trisomy and deletion of one allele out of the three *TP53* alleles; (**D**) chromosome 17 tetrasomy; (**E**) chromosome 17 tetrasomy and deletion of one allele out of the three *TP53* alleles. The following probes were used: Vysis *TP53* (red signals)/CEP17 (green signals) for detection of del(17)(p13.1). Total magnification of 1.500×.

The presented study included 129 MM patients (68 males and 61 females). The rs1042522 variant, expression of *TP53* gene, and cytogenetic changes including 17p13.1 *locus* were analyzed (Figure 2). The number of individuals with deletion of 17p13.1 and with chromosome 17 aneuploidies were low: n = 23 and n = 20, respectively. Deletion of 17p13.1 co-occurred with chromosome 17 aberrations. Among these changes we observed chromosome 17 trisomy (n = 13); chromosome 17 tetrasomy (n = 1); chromosome 17 tetrasomy and deletion of two *TP53* alleles (n = 2); chromosome 17 trisomy and deletion of one *TP53* allele (n = 3); chromosome 17 monosomy (n = 1). Due to the small number of particular chromosome 17 mutations, these changes were grouped and analyzed together. The presence of chromosome 17 aberrations did not affect MM risk and outcome.

### 3.1. Frequencies of Alleles and Genotypes and Their Associations with MM Risk

In contrast to healthy blood donors, the group of MM patients was not in Hardy–Weinberg equilibrium (HWE) (Table 3). The PR genotype was associated with lower risk of MM development (OR = 0.44, *p* = 0.004). However, this effect was not observed for the PP genotype and both PP + PR genotypes (Table 4). We did not observe statistically significant differences between allele frequencies among MM patients and healthy blood donors (Table 4).

### 3.2. TP53 Variants and Risk of Death and MM

Due to the small sample size, the PP homozygote was analyzed together with the heterozygote (PR). Univariate Cox analysis showed that patients in ISS stage III had a 2.08-fold (*p* = 0.002) increased risk of death (Table 5). In the case of MM patients with auto-HSCT, a lower risk of death was observed (HR = 0.24, *p* < 0.001). Similar findings were observed in the case of disease relapse or progression in MM patients at stage III according to ISS (HR = 2.22, *p* < 0.001) and with auto-HSCT (HR = 0.34, *p* < 0.001) (Table 5). The multivariate Cox regression analysis confirmed that patients with auto-HSCT had a decreased risk of death and disease relapse or progression (Table 6). In contrast, patients at stage III according to ISS had an increased risk of death and disease relapse or progression. The univariate and multivariate Cox analysis did not show the impact of studied genotypes on the risk of death or disease relapse or progression in MM patients.

### 3.3. Association of Studied Variant and TP53 Expression with Clinical/Laboratory Values

**Figure 3:**
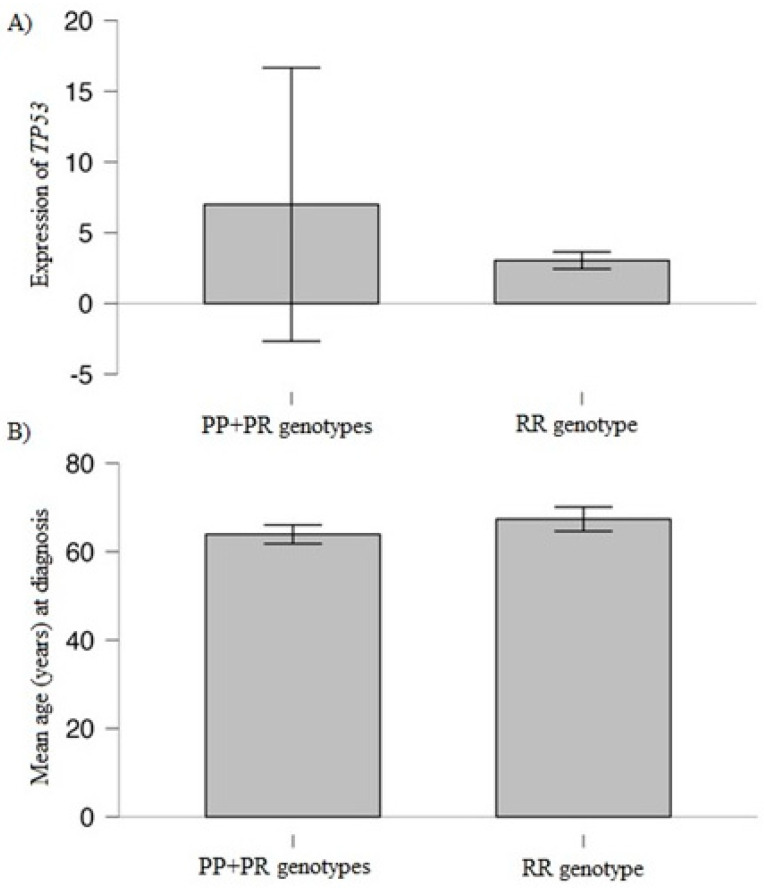
*TP53* genotypes and (**A**) expression level* of *TP53* (*p* < 0.001); (**B**) age of MM patients at diagnosis (*p* = 0.04). *log2 fold change: 6.99 (for PP + PR genotypes) and 3.04 (for RR genotype); delta delta Ct: 5.30 (for PP + PR genotypes) and 13.93 (for RR genotype). In the figure, log2 fold change values are shown.

In the analysis of *TP53* expression, the delta delta Ct method and log2 fold change method were used. We analyzed potential relationships between clinical/laboratory results and selected genotypes, as well as *TP53* expression. Taking into account *TP53* genotypes, we observed a statistically significant higher expression of *TP53* gene in PP + PR genotypes in comparison to RR genotype—PP + PR vs. RR, *p* < 0.001 (Table 7), (Figure 3A). Moreover, we found a higher *TP53* expression in MM smokers in comparison to MM non-smokers—9.86 vs. 8.0, *p* = 0.02. We did not observe statistically significant differences in *TP53* expression taking into account the International Staging System (*p* = 0.65), the presence of another tumor (*p* = 0.79) and exposure to chemicals at work (*p* = 0.06). We observed significant and positive Pearson’s correlation between *TP53* expression and % of plasma cells, r = 0.26, *p* = 0.04. Other correlations between *TP53* expression and clinical data were not significant—for example *TP53* expression and free light chain ratio (*p* = 0.75), number of platelets (*p* = 0.23), concentration of hemoglobin (*p* = 0.58), albumins (*p* = 0.37), creatinine (*p* = 0.59), β2-microglobulin (*p* = 0.20), calcium (*p* = 0.12), C-reactive protein (*p* = 0.79). The presence of the RR genotype was associated with a higher age of MM patients at diagnosis (67.36 vs. 63.91, *p* = 0.04) (Figure 3B), and higher number of plasma cells (36.74 vs. 28.30, *p* = 0.02) in comparison to PP + RR genotypes (Table 7). We did not find an association between other analyzed clinical values and studied genotypes (Table 7).

### 3.4. Survival of MM Patients Taking into Account Type of Treatment and Studied Variants

**Figure 4:**
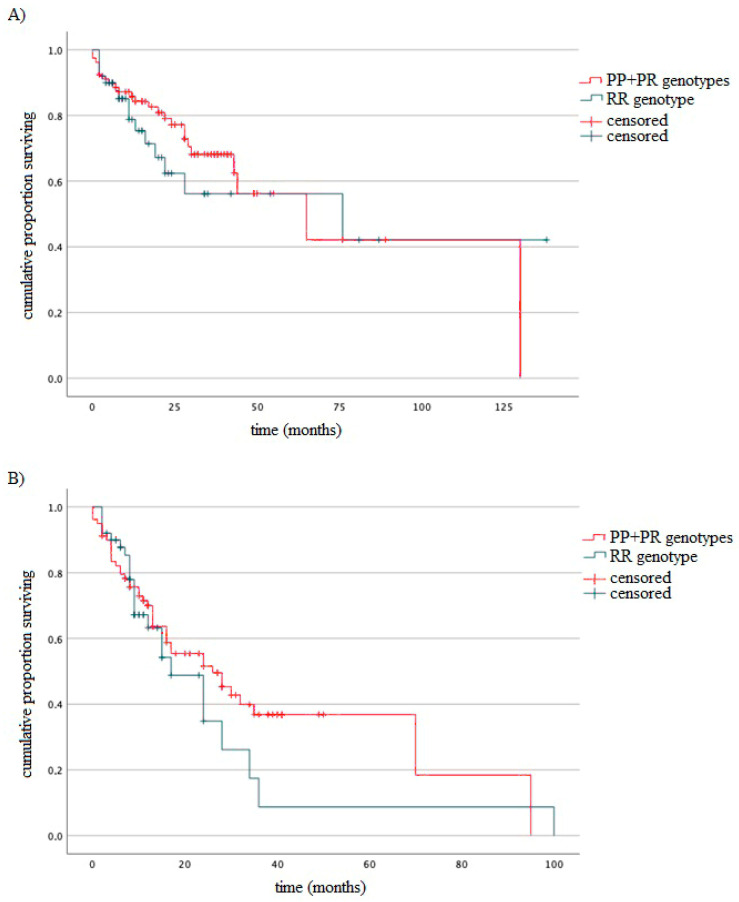
Kaplan–Meier analysis of (**A**) OS (log-rank test *p* = 0.48) and (**B**) PFS (log-rank test *p* = 0.36) taking into account *TP53* genotypes.

The association between studied genotypes and survival of MM patients was analyzed using the log-rank test. Regardless of whether the type of treatment was included or not, we did not observe statistically significant differences in OS and PFS according to TP53 genotypes. Figure 4 shows an example of the OS and PFS analysis including the TP53 variants (without considering the type of treatment).

## 4. Discussion

The detailed role of the p.P72R polymorphism in the etiology and course of multiple myeloma has not been determined in such a wide range. In our study, we have explored the association of the *TP53* rs1042522 variant, expression levels of *TP53* genotypes, deletion of *TP53 locus* and chromosome 17 aneuploidies with the risk of MM development, and response to bortezomib/thalidomide treatment in Caucasian population.

In our study, the PR genotype was associated with a lower risk of MM development, but this effect was not observed for the PP genotype and both PP + PR genotypes. Hattori and colleagues indicated that the *TP53* gene polymorphism of codon 72 did not correlate with the risk of MM [38]. The P allele was associated with earlier relapse and shorter OS in thalidomide therapy [38]. However, their study group was very small (39 patients) [38]. Similarly, studies by Govindasamy et al. showed no association between the p.P72R polymorphism and the risk of MM development [39]. Although in other cancers the p.P72R polymorphism is associated with the etiology of the disease, in the case of MM there is no clear evidence for this.

The meta-analysis by Zhang and colleagues showed no statistically significant association between genotypes of rs1042522 and colorectal cancer risk [40]. Ahmed and coworkers in their research, conducted in the Egyptian population, showed that the R allele was more prevalent among breast cancer patients compared to the control group and that it was not associated with disease progression [41]. Data from Basabaeen et al. suggested that the PP genotype contributed to an increased risk of chronic B lymphocytic leukemia in the Sudanese population ten times more than the RR genotype [42]. Their results indicated a higher frequency of PP homozygotes in the B-CLL patients in comparison to controls. Also, the P allele was associated with a higher risk of leukemia [42]. Chronic lymphocytic leukemia, similarly to MM, originates from malignantly transformed B cells [43]. Dumont et al. reported that the cells with the R allele more effectively induced apoptosis in comparison to cells with the P allele [26]. Their data indicated that the two alleles of *TP53* are functionally distinct, and these differences may influence cancer risk or treatment [26]. In our results, the R allele was associated with a higher age of MM patients at diagnosis. This fact could be connected with a higher potential of R allele cells to induce apoptosis when their physiology processes or DNA functions are disturbed. In this way, potential MM cells were eliminated during apoptosis and the development of malignancy was delayed. However, in our research, a statistically significant higher expression of *TP53* gene in PP + PR genotypes in comparison to RR genotype was observed. But higher expression at the mRNA level does not always mean higher efficiency of the protein. There are miRNAs that directly regulate p53 by binding to the 3′-UTR of the p53 mRNA molecule. The molecules miR-33, miR-125 and miR-504 have been shown to negatively reduce p53 protein levels and promote tumorigenesis [44,45].

We observed significant and positive Pearson’s correlation between *TP53* expression and % of plasma cells. The amount of cDNA transcribed to mRNA used for the qPCR reaction was the same in each sample. *TP53* expression may be upregulated in cancer cells compared to normal cells [46]. Kim et al. showed in their research that the expression of TP53 protein correlates with the variation status of the *TP53* gene in colorectal cancer patients [47]. In our study, we did not test the level of TP53 protein in plasma cells as described in the study limitations.

We found higher *TP53* expression in MM smokers in comparison to MM non-smokers. There are studies showing an association between cigarette smoking and p53 overexpression in esophageal squamous cell carcinoma [48]. Results described by Baosen and coworkers showed that cigarette smoking increased the risk for p53 expression in squamous lung carcinoma. They performed immunohistochemical analysis of p53 expression in lung carcinoma patients [49]. Additionally, Halvorsen and colleagues showed that the frequency of *TP53* mutations in lung cancer patients increases with tobacco consumption [50]. Therefore, it can be hypothesized that continuous exposure to certain carcinogenic components of tobacco smoke, such as PAHs, may cause mutations in some genes regulating the cell cycle, such as *TP53*, leading to overexpression and abnormal accumulation of the protein. Some mutations can result in a dysfunctional protein that is sequestered and accumulated in the cell, contributing to malignant transformation [48,49,50].

In addition to polymorphisms, the attention of researchers is drawn to isoforms of the p53 protein, which may be involved in the course of cancer, e.g., multiple myeloma [51]. So far at least 12 p53 protein isoforms have been identified as a result of alternative splicing, presence of alternative promoters and/or alternative transcription site starts [51,52,53]. Rojas and coworkers found that specific p53 isoform expression is associated with the clinical outcome of MM patients [51]. Therefore, considering the importance of p53 protein in the etiology and course MM, the contribution of mutations, polymorphisms and individual isoforms of the protein should be taken into account to give a more detailed view of MM biology.

The important element in cancer research is the determination of mutations in the *TP53* gene. De Souza et al. checked the effect of missense mutations in the *TP53* gene and the p.P72R polymorphism on the process of carcinogenesis. They showed that if a mutation appears in the *TP53* gene, its effect depends on which allele the P or R occurs on [20]. They showed that missense mutations present in R alleles lead to carcinogenesis because general function of the protein is lost. Mutated P alleles are negatively selected for carcinogenesis because p53 will still bind to chromatin sites and induce a gene expression program consistent with maintenance of wild-type p53 function [20]. This means that the oncogenic effect of mutated *TP53* is inhibited by the function of the P allele [20].

In summary, research on the role of the *TP53* gene and its protein in the pathogenesis and course of MM should be comprehensive and should include mutations, polymorphisms, gene expression, protein level and its isoforms. In addition, it is important to use methods that allow the detection of subclonal changes because it is a main factor for the prognostication of the disease course. Only comprehensive molecular analysis will allow for precise determination of the *TP53* gene and protein role in the etiology and course of MM.

A limitation of our study is the relatively small sample size in part due to the low incidence of MM. Further analysis with a larger cohort can help better understand the significance of the *TP53* rs1042522 variant in the pathobiology of MM. Other limitations were associated with the lack of point mutations’ determination in the *TP53* gene using the next generation sequencing method. This would enable us to assess the relationship between the analyzed variants and the presence of mutations in this gene. We did not evaluate the *TP53* expression at the protein level due to the low number of plasma cells obtained after magnetic isolation. Taking into account the epigenetic mechanisms’ high expression at the mRNA level does not mean high expression at the protein level. It is a missing “puzzle piece” in our research.

## 5. Conclusions

*TP53* gene expression and its rs1042522 variant did not affect the MM outcome. We have shown that the p.R72R variant was associated with a higher age of MM patients at diagnosis. We observed higher *TP53* expression in MM smokers in comparison to MM non-smokers. In the future, the study group should be enlarged, and the research extended to include the analysis of *TP53* expression at the protein level.

## Figures and Tables

**Table 1:** The characteristics of MM patients.

Variables	MM Patients, n = 129
Age	65.24 (mean)
Sex	
Male	68
Female	61
Type of MM	
IgG	72
IgA	28
Light chain	29
Stage according to the International Staging System	
I	32
II	37
III	60
Smoking	
Yes	20
No: Non-smokers	97
No: Ex-smokers	12
Exposure to carcinogenic factors	
Yes	26
No	103
Additionally other type of cancer	
Yes	8
No	121
Renal failure	
Yes	102
No	27
The stage of chronic kidney disease (grade)	
G1	39
G2	36
G3A	18
G3B	15
G4	10
G5	11
Anemia grade before treatment (WHO)	
Absent	30
I—mild	43
II—moderate	44
III—severe	12
Structural cytogenetic changes	
del(17p13.1)	15
del(17p13.1) and t(4;16)	7
del(17p13.1) and t(14;16)	1
t(4;14)	12
t(14;16)	3
Chromosome 17 aneuploidies	
Present	20
Absent	109
Chemotherapy	
CTD ^1^	49
VCD ^2^ and VD ^3^	43
VTD ^4^	35
Died before chemotherapy	2

^1^ Cyclophosphamide, Thalidomide and Dexamethasone; ^2^ Velcade, Cyclophosphamide and Dexamethasone; ^3^ Velcade and Dexamethasone; ^4^ Velcade, Thalidomide and Dexamethasone.

**Table 2:** Inclusion and exclusion criteria for MM patients and healthy blood donors.

Inclusion Criteria for MM Patients and Healthy Blood Donors	Exclusion Criteria for MM Patients
- Signed informed consent. - 18 years of age or older. - Successful genotyping.	- Active smoldering MM. - Active plasma cell leukemia. - Documented systemic amyloid light chain amyloidosis. - Active central nervous system involvement with MM.
Additional inclusion criteria for MM patients	Exclusion criteria for healthy donors
- Newly diagnosed disease. - Measurable disease for secretory, poor secretory and non-secretory MM. For secretory MM, the presence of quantifiable monoclonal component, ≥0.5 g/dL, was taken into account. For poor secretory or non-secretory MM, the level of the affected serum free light chain must be ≥10 mg/dL or ≥100 mg/L with an abnormal free light-chain ratio. - Eastern Cooperative Oncology Group (ECOG) Performance status ≤3. - Life expectancy more than 3 months.	- HIV infection, hepatitis B or C infection. - Bacterial infection resulting in the development of syphilis or tuberculosis. - Severe coagulation disorders (e.g., hemophilia) or significantly impaired venous access. - A condition that requires active medical intervention or monitoring to avert serious danger to the participant’s health or well-being.

**Table 3:** Hardy–Weinberg equilibrium (HWE) for the *TP53* variant in the case and control groups according to expected (E) and observed (O) values.

Groups	Genotypes of *TP53*—p.P72R Variants			Total	HWE *p* Value and χ^2^ *
-	PP	PR	RR	-	-
Control					
E	9.92	43.15	46.92	100	*p* = 0.07 χ^2^ = 3.17
O	15	33	52	100	
Case					
E	14.33	57.33	57.33	129	*p* = 0.03 χ^2^ = 4.68
O	7	72	50	129	

* If the χ^2^ ≤ 3.84 and the corresponding *p* ≥ 0.05, then the population is in HWE.

**Table 4:** The comparison of allele frequency and distribution of the *TP53* variant among MM patients and controls.

Gene Variants and Alleles	MM, n (%)	Controls, n (%)	Odds Ratio	95% Cl	*p* Values
RR	50 (38.76%)	52 (52%)	1	-	-
PR	72 (55.81%)	33 (33%)	0.44	0.25–0.77	0.004
PP	7 (5.42%)	15 (15%)	2.06	0.77–5.47	0.14
PP + PR	19 (18.81%)	14 (14%)	0.70	0.32–1.56	0.39
Total	129 (100%)	100 (100%)			
R	173 (67%)	134 (67%)	1	-	-
P	85 (33%)	66 (33%)	1	0.67–1.47	1.0
Total	258 (100%)	200 (100%)			

**Table 5:** Univariate Cox analysis in survival of MM patients.

Variable	Univariate Cox Analysis for OS			Univariate Cox Analysis for PFS		
	*p* Value	HR	95% Cl	*p* Value	HR	95% Cl
ISS						
I + II	-	R		-	R	-
III	0.002	2.08	1.30–3.32	<0.001	2.22	1.26–2.43
Auto-HSCT						
Yes	<0.001	0.24	0.10–0.57	<0.001	0.34	0.19–0.61
No	-	R	-	-	R	-
*TP53* P72R variant						
RR	-	R	-		R	
PR + PP	0.49	1.26	0.65–2.43	0.37	1.27	0.75–2.14

**Table 6:** Multivariate Cox analysis in survival of MM patients.

Variable	Multivariate Cox Analysis for OS			Multivariate Cox Analysis for PFS		
	*p* Value	HR	95% Cl	*p* Value	HR	95% Cl
ISS						
I + II	-	R		-	R	-
III	0.04	1.66	1.02–2.70	0.03	1.46	1.03–2.05
Auto-HSCT						
Yes	0.01	0.31	0.13–0.77	0.005	0.40	0.22–0.76
No	-	R	-	-	R	-
*TP53* P72R variant						
RR	-	R	-	-	R	-
PR + PP	0.51	1.25	0.65	0.24	1.38	0.81–2.35

**Table 7:** The clinical values of MM patients taking into account studied variants.

Variables	MM Patients	*TP53* rs1042522		
		PP + PR	RR	*p*-Value
Mean age (years) *	65.24	63.91	67.36	0.04
*TP53* expression	8.35	7.0	3.04	*p* < 0.001
Free light chain ratio *	252.43	290.45	192.38	0.41
% of plasma cells in bone marrow *	32	28.30	36.74	0.02
Albumins (g/dL) *	3.62	3.64	3.56	0.78
β2-microglobulin * (mg/L)	6.81	6.82	6.77	0.74
Calcium * (mM/L)	2.46	2.51	2.43	0.43
Hemoglobin * (g/dL)	10.29	10.29	10.28	0.88
Creatinine * (mg/dL)	1.63	1.41	1.94	0.27
Platelets (K/μL)	214.71	223.92	200.16	0.27
C-reactive protein * (mg/L)	14.36	14.88	13.30	0.24
Estimated glomerular filtration rate * mL/min/1.73 m^2^	67.67	70.72	62.97	0.19

* At diagnosis.

## Data Availability

The clinical data used to support the findings of this study are available from the corresponding author upon request.
